# Nivolumab for Recurrent or Metastatic Head and Neck Squamous Cell Carcinoma: A Retrospective Tertiary Centre’s Real-World Experience

**DOI:** 10.3390/curroncol30100645

**Published:** 2023-09-29

**Authors:** Yue (Jennifer) Du, Rui Fu, Justin T. Levinsky, Pabiththa Kamalraj, Kelvin K. W. Chan, Ambica Parmar, Antoine Eskander, Martin Smoragiewicz

**Affiliations:** 1 Temerty School of Medicine, University of Toronto, Toronto, ON M5S 1A8, Canada; jennifer.du@medportal.ca; 2 Department of Otolaryngology—Head and Neck Surgery, Sunnybrook Health Sciences Centre, Toronto, ON M4N 3M5, Canada; rui.fu@mail.utoronto.ca (R.F.); jlevins2@uwo.ca (J.T.L.); pabiththa.kamalraj@sri.utoronto.ca (P.K.);; 3 Odette Cancer Centre, Sunnybrook Health Sciences Centre, Toronto, ON M4N 3M5, Canada; kelvin.chan@sunnybrook.ca (K.K.W.C.); ambika.parmar@sunnybrook.ca (A.P.)

**Keywords:** nivolumab, head and neck, squamous cell carcinoma, cancer, chemotherapy, PD-1 checkpoint inhibitor

## Abstract

Nivolumab, a PD-1 checkpoint inhibitor, was approved in Canada in 2017 for the treatment of recurrent or metastatic head and neck squamous cell carcinoma (R/M HNSCC) based on the phase 3 trial CHECKMATE-141. We aimed to examine the demographics and efficacy of nivolumab in a Canadian, real-world setting. A retrospective chart review was performed on patients who received nivolumab for R/M HNSCC from 2017 to 2020 at a high-volume cancer centre. Data were abstracted from 34 patients, based on physician notes and imaging reports. The median patient age at nivolumab initiation was 61, 24% were female, and 62% were current or former smokers. Prior to nivolumab, 44% of patients underwent surgery, 97% radiation, and 100% chemotherapy. Most (97%) therapies were for primary disease. Overall survival at 6 and 12 months following drug initiation was 38% and 23%, respectively. Progression-free survival at 6 and 12 months was 33% and 22%, respectively. Eighteen percent of patients experienced an immune-related adverse event, the most common of which was pneumonitis (3/8) and endocrine events (3/8). Seven out of eight of the immune adverse events were grade 1–2; 1/8 was grade 3. Nivolumab appears to have decreased survival rates in our single-centre Canadian population compared to CHECKMATE-141 and presented a manageable adverse event profile for R/M HNSCC.

## 1. Introduction

Sixty percent of patients with head and neck squamous cell carcinoma (HNSCC) present with locoregionally advanced disease that is treated with either surgery or radiation, often in combination with chemotherapy [1,2,3,4]. Unfortunately, there is a high rate of local, regional, or metastatic disease recurrence (20–30%) and only a minority of these patients are eligible for potentially curative salvage treatment [1,4,5,6]. Prior to the availability of pembrolizumab in the first-line setting in Canada in 2021, most patients with incurable recurrent or metastatic HNSCC (R/M HNSCC) would be considered for palliative-intent first-line platinum-based chemotherapy, with or without cetuximab [2,5]. Upon progression, there were a small number of options for management of these patients with poor outcomes [1,6].

The PD-1 inhibitor nivolumab was approved in 2017 in Canada for the treatment of R/M HNSCC following platinum-based chemotherapy or recurrence within 6 months of definitive chemoradiation [7]. The phase 3 trial CHECKMATE-141 demonstrated improved overall survival (OS) with a reasonable safety profile for second-line treatment [3]. However, the real-world use of nivolumab and its effectiveness remain unclear, especially in the Canadian context. We aimed to examine the demographics of patients prescribed nivolumab, radiographic response, OS, and immune-related toxicity in a real-world setting at a tertiary care head and neck designated cancer centre.

## 2. Materials and Methods

### 2.1. Study Design and Cohort

A retrospective chart review was performed on patients who received nivolumab for R/M HNSCC from 2017 to 2020 at Sunnybrook Health Sciences Centre, a high-volume academic cancer centre. This study was approved by the research ethics board at Sunnybrook Health Sciences Centre (REB number: 2217). Key eligibility criteria included an age of onset of at least 18 years old, receiving nivolumab for R/M HNSCC, having HNSCC confirmed via initial histology, and experiencing recurrent or progressive cancer within 6 months of their last dose of platinum-containing chemotherapy for primary or recurrent HNSCC. Individuals were excluded if they received systemic treatment for any other primary cancer within 6 months of their first dose of nivolumab.

### 2.2. Data Collection

Key demographic and clinical characteristics were extracted from the medical records of eligible patients and verified by 2 researchers (JD, MS), including primary tumor location, metastasis location, histologic type, cancer stage at diagnosis, p16 status, nivolumab treatment history, subsequent treatments, date of death, and cause of death. Best imaging response to treatment was abstracted from clinical radiology reports but was not quantified using RECIST [8]. Standard-of-care imaging was usually performed using computed tomography (CT) every 3 months. Immune-related adverse events (irAEs) were retrospectively graded according to CTCAE v5.0.

### 2.3. Outcomes

The objective response rate (ORR) was defined as a partial or complete radiographic response on imaging following nivolumab administration by the authors retrospectively. The irAE rate was defined as the proportion of patients experiencing pneumonitis, hepatitis, colitis, endocrine events, or other irAEs related to nivolumab. Overall survival (OS) time was defined as the time from the first nivolumab infusion to date of death. Patients who were alive at the end of the study or became lost to follow-up were censored. Progression-free survival (PFS) time was the time from the first nivolumab infusion to the date of documented disease progression or the date of death, whichever came first. Patients who did not experience disease progression and were alive (or lost to follow-up) were censored.

### 2.4. Statistical Analysis

Patient characteristics were described with median and interquartile range (IQR) or count and frequency (for categorical variables). For response and irAE rates, we reported the total number of events observed and stratified by grade.

For OS and PFS, we assessed them separately in a time-to-event analytical framework using the Kaplan–Meier approach. Specifically, we estimated the median survival time and the associated 95% confidence interval (CI), which indicated the length of time from nivolumab initiation that half of the cohort was event-free. Probabilities of OS and PFS at 6 and 12 months post nivolumab initiation were estimated. All statistical analyses were 2-sided where the *p*-value < 0.05 was set to indicate statistical significance. All analyses were performed using R version 3.6.1 (R Foundation for Statistical Computing).

## 3. Results

### 3.1. Demographic Characteristics

From 2017 to 2020, 34 patients were included in the study (Table 1). The median patient age at nivolumab initiation was 61 years and most patients were male (26/34, 76%). Most patients were current (10/34, 29%) or former smokers (11/34, 32%). Nivolumab was administered at 6 mg/kg (up to a maximum of 480 mg) through IV every 4 weeks. The median follow-up time was 9.4 (interquartile range (IQR), 4.7–14.3) months from the date of cancer recurrence diagnosis or 4.6 (IQR 2.2–9.2) months from the date of nivolumab initiation, leaving a median time gap of 2.0 (IQR 0.7–6.4) months between diagnosis and the first dose of nivolumab. One patient (3%) was lost to follow-up due to moving overseas.

### 3.2. Clinical Characteristics

The most common primary site was the oral cavity (16/34, 47%) and oropharynx (11/34, 32%). Of the 11 oropharynx patients who underwent p16 testing, 7 (64%) were positive. All patients were initially non-metastatic, but 31 out of 34 patients had recurrences with distant metastases (65%), primarily in the lungs (53%) and bones (18%) but also to the liver (9%), mediastinum (6%), and other locations (18%).

### 3.3. Treatment History

Prior to nivolumab initiation, 15 (44%) patients underwent surgery, 33 (97%) patients received definitive radiation, and all 34 (100%) patients underwent platinum-based chemotherapy. In terms of prior treatments, 21 (62%) patients received primary chemoradiation, 11 (32%) received primary surgery, 1 (3%) patient received primary radiation, and 1 (3%) patient received primary chemotherapy. In total, 33 (97%) of the prior treatments were used for primary disease, 10 (29%) were used for metastatic disease, and 1 (3%) was used for neoadjuvant disease. A total of 21 (62%) patients received one previous line of systemic therapy, 12 (35%) patients received two previous lines, and 1 (3%) patient received four previous lines of systemic therapy. Following nivolumab treatment, 24% of patients received subsequent chemotherapy and 6% received subsequent radiation.

### 3.4. Efficacy

Five (15%) patients’ responses to treatment were not reassessed via CT. As such, we excluded these five patients from the PFS analysis. Among those who underwent at least one CT assessment (n = 29, 85%), we observed 9/29 partial responses (31%, 95% CI: 17–50%), 16/29 disease progressions (55%, 95% CI: 38–72%), and 4/29 patients had stable disease as a best response (14%, 95% CI: 6–31%).

### 3.5. Survival

**Figure 1:**
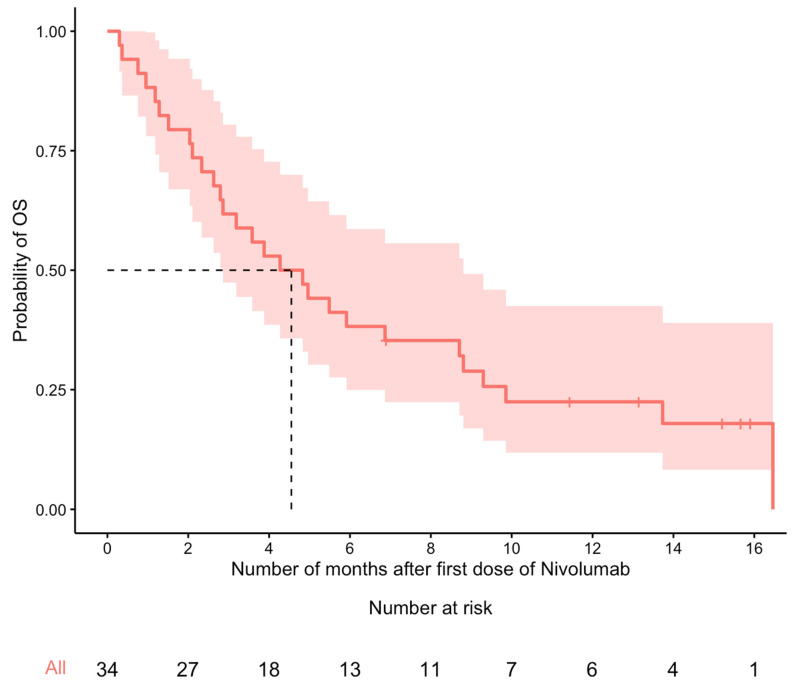
Kaplan–Meier curve for OS (n = 34). The median OS time (dashed line) was 4.6 months (95% confidence interval: 2.9–8.8 months).

**Figure 2:**
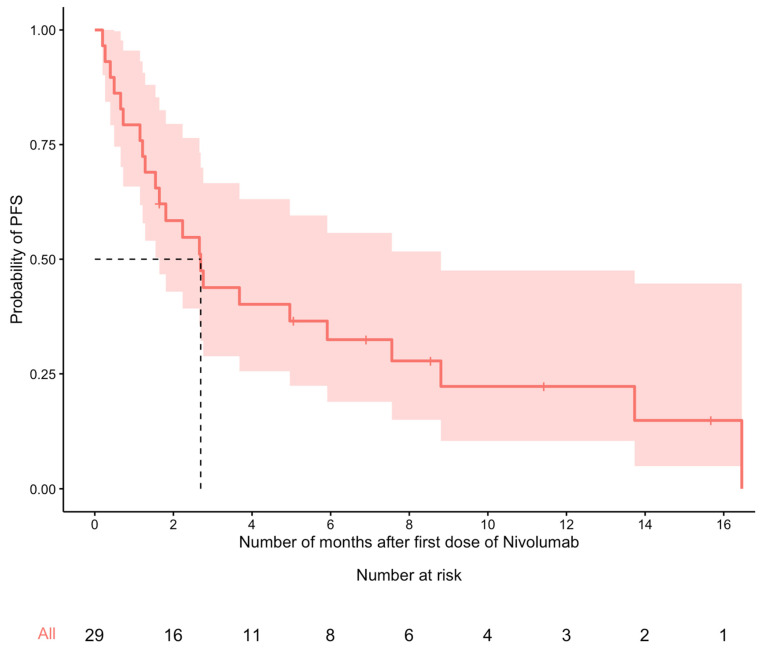
Kaplan–Meier curve for PFS (n = 29). The median PFS time (dashed line) was 2.7 months (95% confidence interval: 1.6–8.8 months).

At the end of the study period, 28 (82%) patients had died. Median OS was 4.6 months (95% CI: 2.9–8.8). Using the Kaplan–Meier method, the estimated OS probability at 6 and 12 months was 38% (95% CI: 25–59%) and 23% (95% CI: 12–43%), respectively (Figure 1). Among the 29 patients with complete disease progression data, the median PFS time was 2.7 months (95% CI: 1.6–8.8). The estimated PFS probability at 6 and 12 months was 33% (95% CI: 19–56%) and 22% (95% CI: 10–48%), respectively (Figure 2).

### 3.6. Adverse Events

Six (18%) patients experienced a total of eight irAEs. Three patients experienced pneumonitis, three patients experienced hypothyroidism, one patient developed colitis, and one patient experienced a flare of rheumatoid arthritis. Among the eight irAEs, seven (88%) were grade 1 or 2, and there was one colitis case (13%) that was grade 3 (Table 2).

## 4. Discussion

From our population of 34 patients at a Canadian tertiary care centre, the majority of patients who received nivolumab were males, had a smoking history, and typically had an oral cavity or oropharynx primary at an advanced cancer stage at diagnosis. Most patients had received multiple modalities of cancer treatment (surgery 44%, chemotherapy 100%, and radiation 97%) prior to nivolumab initiation. Only 29% of patients received systemic therapy for metastatic disease before receiving nivolumab (vs. 46.7% in CHECKMATE-141), likely reflecting a higher proportion of patients relapsing within 6 months of definitive chemoradiation treatment and overall poorer prognosis. Finally, patients were also found to be mostly ECOG 0 and 1, but a few patients had a higher symptom burden with ECOGs of 2–3, which differs from the eligibility criteria form CHECKMATE-141 [3].

Given the apparent differences in these patient characteristics compared to the CHEKMATE-141 study, it is perhaps not surprising that our real-world study suggests lower real-world effectiveness with lower median OS (7.5 vs. 4.6 months) and 1-year landmark OS (36% vs. 23%) [3]. Our observed response rate to nivolumab was 31.0% (95% CI: 17.3–49.2%) which is higher than that of the CHECKMATE-141 trial that reported a response rate of 13.3% (95% CI: 9.3–18.3%). It is important to note that our response rate is not RECIST-defined due to the retrospective nature of the study and lack of reporting on the percentage of tumor volume reduction [8]. As such, a radiologist may have reported a response rate that may not have met the 30% decrease in tumor volume required for a RECIST-defined response [8]. IrAEs were experienced in 17.7% of patients, were mainly low grade, and comparable to 13.1% in CHECKMATE-141 [3].

These findings support the continued use of nivolumab in this population but identify the need for better treatment regimens in the recurrent metastatic setting of HNSCC. This is particularly true given that our population had worse ECOG scores than would be typically accepted in a clinical trial [3,9]. Other studies of real-world-setting nivolumab use are consistent with ours in terms of efficacy and adverse events [3,9,10,11]. This includes one group in England and one group in Japan which found a response rate to nivolumab of around 13–20% of patients with 1-year survival rates between 28 and 41% [3,9,10,11]. One group in England studied real-world outcomes of nivolumab treatment for R/M HNSCC in 123 patients from four large cancer centres in England [11]. They reported an overall response rate of 19.3% and one-year OS of 28.6% with a minimal side effect profile with 15.1% of patients experiencing therapy side effects [11]. Another large-centre study was conducted examining 100 patients from multiple institutions in Japan who received nivolumab for R/M HNSCC [12]. They found an overall response rate of 13.5% with a median OS of 9.6 months. IrAEs occurred in 30% of the patients in Japan [12]. Interestingly, they found that the one-year OS rate was better in patients who developed irEAs compared to those who did not (52.67% to 34.0%, respectively) [12]. Our patient numbers were too low to statistically test for this [12]. Another Japanese group investigated 52 patients with R/M HNSCC and found a one-year OS rate of 40.4% [4]. This group specifically investigated PD-L1 expression and found that high expression of PD-L1 (≥40%) was associated with improved OS, which speaks to the benefit of personalized treatment options for future practice [4].

With the rise in the number of immunomodulating therapies for the treatment of many cancers, there has been a need to balance potential benefits with adverse effects. IrAEs were experienced in 18% of our patients compared to 13.1% in CHECKMATE-141 [1]. We identified that pneumonitis and endocrine events were the most common IrAEs in our cohort. Although most of our reported adverse events were low grade, all patients who experienced an IrAE stopped their treatment course. One group investigating the side effect profile of immunomodulating therapies found that the treatment of IrAEs mostly consisted of therapy cessation, supportive therapy, and sometimes a course of steroids [2]. Although most patients had a complete resolution of IrAEs, some developed long-term adverse effects [2]. The risks and benefits of therapy will be weighed on a patient-specific basis.

Limitations of our study include the small sample size from a single centre and the study’s retrospective nature. Additionally, our response assessment was based on chart-reported assessments rather than the RECIST guideline. The strengths of this study include data collection from a recent cohort and examining a real-world population from a tertiary care centre in a highly regionalized cancer system. Population data will take time to collect, and increasing the number of studies from multi-centre patient populations treated with nivolumab will continue to identify how this systemic therapy is functioning and which patients might benefit most from its use.

## 5. Conclusions

Our research group and groups in Japan and England demonstrated that while nivolumab appears to work for a small group of patients in real-world settings, it is far from a universally efficacious treatment [10,12]. New treatments are needed to improve the outcomes of patients with R/M HNSCC. Pembrolizumab is now available for first-line treatment of R/M HNSCC, and the use of nivolumab will likely decrease [13,14]. However, it will remain a treatment option for patients who have recurrences within 6 months of definitive chemoradiation [13,14]. OS is still poor, and further research should explore other therapeutic options for this group [3,4,11,12]. Future studies should explore the bioselection of patients for different systemic treatment options [4].

## Figures and Tables

**Table 1:** Demographic and clinical characteristics.

Demographic Characteristics	n (%)
Age at diagnosis, median (IQR)	61 (36–76)
Males	76
Smoking History	
Current	10 (29%)
Former	11 (32%)
Never	13 (38%)
Primary Tumour Site	
Oral cavity	16 (47%)
Oropharynx	11 (32%)
Nasopharyngeal	1 (3%)
Larynx	1 (3%)
Hypopharynx	3 (9%)
Other (combinations of the above)	2 (6%)
Sites of Metastasis	
Lung	18 (53%)
Bone	6 (18%)
Liver	3 (9%)
Mediastinum	2 (6%)
Other	6 (18%)
p16 Status	
Positive	7 (21%)
Negative	6 (18%)
Not tested	21 (62%)
Stage Group at Locally Advanced/Metastatic Diagnosis	
Stage 3	3 (9%)
Stage 4	31 (91%)
ECOG Performance-Status Score	
ECOG 0	6 (26%)
ECOG 1	14 (61%)
ECOG 2	3 (13%)
ECOG ≥ 3	1 (4%)
Number of Previous Lines of Systemic Therapy	
1	21 (62%)
2	12 (35%)
≥3	1 (3%)
Context of Prior Systemic Therapy	
Primary disease	33 (97%)
Neoadjuvant therapy	1 (3%)
Metastatic disease	10 (29%)

ECOG score: Eastern Cooperative Oncology Group score; IQR: interquartile range; n = number of individuals.

**Table 2:** IrAEs.

Type of IrAE	No. Adverse Events/Total No. Adverse Events
**Pneumonitis**	
Grade 1	3/8
**Endocrine**	
Grade 1	1/8
Grade 2	2/8
**Colitis**	
Grade 3	1/8
**Other**	
Grade 2	1/8

IrAE: immune-related adverse event; No.: number.

## Data Availability

Data are not publicly available.
